# Antibacterial Coating Based on Functionalized MoS_2_ Quantum Dots

**DOI:** 10.3390/ma18061352

**Published:** 2025-03-19

**Authors:** Toby Chan, Soha Ahmadi, Zahra Ramezani, Michael Thompson

**Affiliations:** 1 Department of Chemistry, University of Toronto, 80 St George St, Toronto, ON M5S 3H6, Canada; tobywaiho.chan@mail.utoronto.ca (T.C.); soha.ahmadi@mail.utoronto.ca (S.A.); zahramezani@gmail.com (Z.R.); 2 Toxicology Research Center, Medical Basic Sciences Research Institute, Ahvaz Jundishapur University of Medical Sciences, Ahvaz 6135715794, Iran

**Keywords:** quantum dots, antibacterial, hydrothermal synthesis, *Staphylococcus aureus*

## Abstract

MoS_2_ quantum dots (QDs) were synthesized using a one-step hydrothermal method and subsequently functionalized with 11-mercaptoundecanoic acid. The functionalized QDs were thoroughly characterized, which exhibited antibacterial activity against *Staphylococcus aureus* at 10 mg/mL. These findings underscore its potential as antifouling coatings for biomedical applications.

## 1. Introduction

Quantum dots (QDs) have gained widespread attention for their unique electrical and optical properties [1]. However, a more recent and promising application lies in their use for antimicrobial purposes, as QDs have been found to not exhibit the same effects of antibacterial resistance as antibiotics. Studies investigating the antibacterial mechanisms for functionalized QDs have identified the following three primary molecular pathways: (1) the destruction or inhibition of the cell membrane/cell wall, (2) the generation of reactive oxygen species (ROS), leading to cellular damage, and (3) the binding with DNA/RNA to inhibit cellular reproduction [2]. These mechanisms highlight the versatility of QDs in combating bacterial infections.

Bacterial infections in healthcare settings are a significant concern, responsible for over 16 million additional hospital days annually spent in Europe, substantially increasing healthcare costs [3]. Moreover, the overuse of antibiotics for treatment has worsened the problem, contributing to the emergence of multidrug-resistant pathogens. These resistant pathogens, particularly bacteria, are also a major cause of mortality, accounting for an estimated 700,000 deaths globally each year. In this context, QDs present a promising alternative, with the potential to address some challenges associated with bacterial resistance to broad-spectrum antibiotics [4].

Among the various QDs, molybdenum disulfide (MoS_2_), a transition metal dichalcogenide (TMDC), has gained particular interest. MoS_2_ is generally non-cytotoxic and thus biocompatible for use in medical applications, such as implants or biomedical instruments such as dialysis machines [5].

In this study, a one-step process involving lithium intercalation in conjunction with hydrothermal treatment was used to synthesize MoS_2_ quantum dots (MoS_2_ QDs) from bulk MoS_2_ powder. This approach offers a cost-effective, environmentally friendly, and time-efficient method for QD synthesis. The bare MoS_2_ QDs were subsequently functionalized with 11-mercaptoundecanoic acid (11-MUA) to produce functionalized MoS_2_ QDs (F-MoS_2_ QDs). Detailed synthesis procedures are provided in the Supplementary Materials.

## 2. Materials and Methods

The hydrothermal synthesis method used bulk MoS_2_ powder in a 10% ethanol solution, processed for 24 h. Using a conventional oven method, the exfoliation of MoS_2_ QDs from the bulk powder achieved a yield of approximately 10%. Extending the reaction time did not improve the yield. Additional attempts to improve the yield through alternative methods, such as synthesis from bulk sodium molybdate using L-cysteine, were unsuccessful (see Figure S1). Detailed protocols for these alternate methods are also described in the SI. The bulk material, bare MoS_2_ QDs, and F-MoS_2_ QDs were characterized using fluorescence microscopy, UV–Vis, fluorescence, IR spectroscopy, Raman spectroscopy, dynamic light scattering (DLS), and zeta potential analysis.

## 3. Results and Discussion

**Figure 1:**
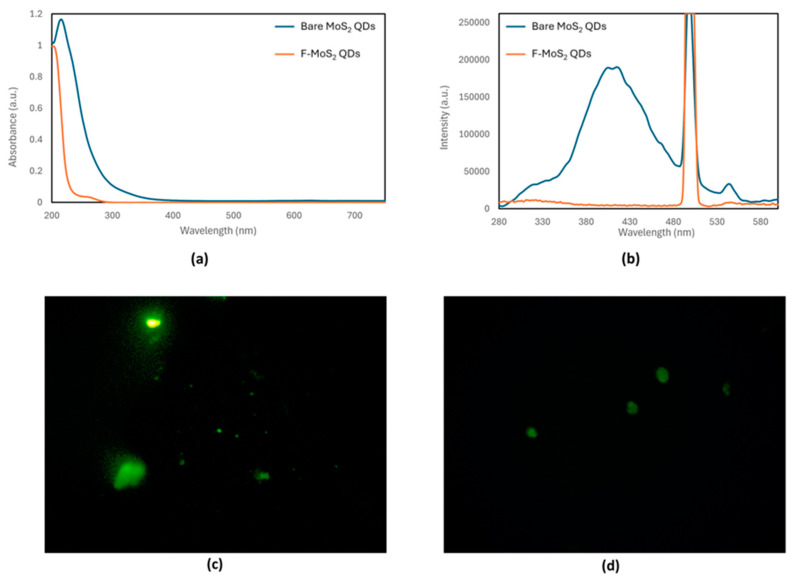
(**a**) UV–Vis spectra, (**b**) fluorescence spectra, and fluorescence microscopy images: (**c**) green emission of bare MoS_2_ QDs and (**d**) F-MoS_2_ QDs.

Both the bare MoS_2_ QDs and F-MoS_2_ QDs exhibited fluorescence, emitting green when exposed to light using the blue excitation filter under the epifluorescence microscope, with notable morphological differences (Figure 1c). The bare MoS_2_ QDs appeared irregular in shape, likely due to their hygroscopic nature, which led to the formation of solution droplets upon exposure to air. In contrast, the F-MoS_2_ QDs displayed a more defined ellipsoid shape with solid edges. These observations suggest that functionalization enhances the stability of the QDs, resulting in a more uniform shape and size compared to the bare QDs.

The UV–Vis absorbance spectra of the bare MoS_2_ QDs and F-MoS_2_ QDs exhibited notable differences (Figure 1b). The absorbance maximum of the F-MoS_2_ QDs showed a blue shift of 10 nm compared to that of the bare MoS_2_ QDs, shifting from 215 nm to 205 nm. Furthermore, the F-MoS_2_ QDs displayed a minor shoulder peak around 280 nm, likely due to the absorption by 11-MUA.

Fluorescence spectra were recorded at different excitation wavelengths to determine the optimal excitation wavelength for the MoS_2_ QDs, which was determined to be 250 nm. This suggests a direct fluorescent transition between the valence and conduction bands. The presence of an emission peak confirmed the successful exfoliation of the MoS_2_ QDs from the bulk material. Furthermore, the fluorescence emission spectrum indicates that the synthesized QDs are likely less than 50 nm in diameter, as the bulk material does not fluoresce [6]. In contrast, the F-MoS_2_ QDs do not exhibit any fluorescent emission, likely due to the quenching caused by the introduction of 11-MUA as a functionalizing chemical. This observation aligns with findings by Chen, W.-Y. et al., who also reported the quenching of 11-MUA functionalized quantum dots by O_2_ in aqueous solutions [7].

As shown in Figure S2, the IR spectrum of the bulk material exhibits several poorly resolved peaks, consistent with the IR spectra reported in the literature [8]. The broad hydroxyl (O-H) stretching peak indicates water content within the bulk material. These results confirm that the bulk MoS_2_ powder used in this study is similar to those employed in other hydrothermal synthesis experiments, as reported in previous studies [8,9].

**Figure 2:**
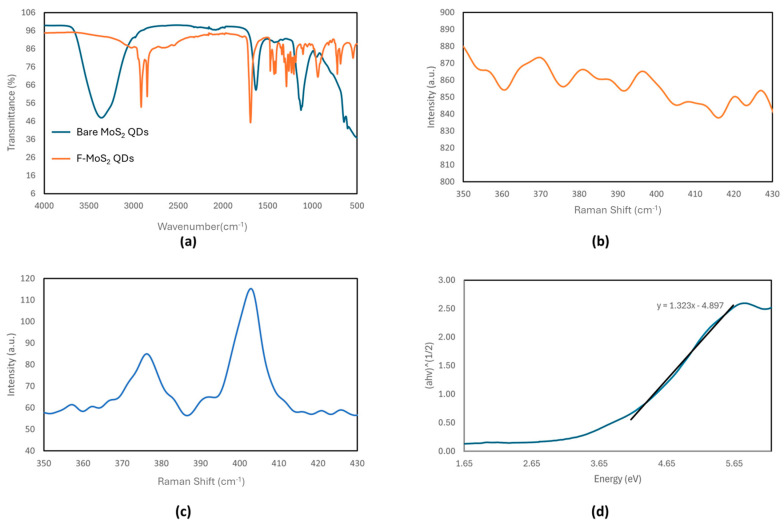
(**a**) IR spectra of the bare MoS_2_ QDs and F-MoS_2_ QDs, Raman spectra of (**b**) the bare MoS_2_ QDs and (**c**) bulk MoS_2_, and the (**d**) Tauc plot of the bare MoS_2_ QDs.

In contrast, the IR spectrum of the bare MoS_2_ QDs (Figure 2b) differs significantly from that of the bulk powder. Characteristic peaks, such as the one at 1630 cm^−1^ corresponding to S=O and the one at 605 cm^−1^ corresponding to Mo-S, indicate the successful exfoliation of QDs from the bulk MoS_2_ powder, which is consistent with the literature reports [9,10]. The broad O-H stretching peak at 3300 cm^−1^ likely arises from the hygroscopic nature of the sample, which absorbs moisture from the air. Additionally, the peak at 1126 cm^−1^, characteristic of S=O stretching from sulfate groups, is consistent with data from Hariharan et al. [11]. The significant spectral differences between the QDs and the bulk material can be attributed to variations in the surface functional groups, such as the potential presence of sulfate groups on the single-layer QDs.

As shown in Figure S3, the spectrum of the F-MoS_2_ QDs reveals no characteristic peaks of phenylalanine, suggesting that functionalization via EDC-NHS coupling was unsuccessful. However, functionalization with 11-mercaptoundecanoic acid (11-MUA) was successful, as evidenced by the appearance of C-H and C=O bond stretching peaks. Furthermore, the absence of the O-H stretching peak in the F-MoS_2_ QDs indicates increased stability under ambient conditions compared to the bare MoS_2_ QDs.

Raman spectroscopy is a common technique used to elucidate the layered structure of TMDC materials like MoS_2_. The Raman spectrum of bulk MoS_2_ is well documented, with the following two prominent Raman-active modes associated with Mo-S bonds: the in-plane vibrational (E^1^_2g_) band at 385 cm^−1^ and the out-of-plane vibrational (A_1g_) band at 405 cm^−1^. As shown in Figure 2c, the Raman spectrum of the bulk MoS_2_ in this study exhibits these two characteristic peaks, consistent with the previously reported data [12,13].

In contrast, the Raman spectra for the bare MoS_2_ QDs (Figure 2b) lack resolvable peaks compared to the bulk MoS_2_ spectra (Figure 2c), which exhibit characteristic Raman peaks. As such, the Raman spectra of the F-MoS_2_ QDs do not have any present E^1^_2g_ or A_1g_ peaks (Figure 2b). This observation is consistent with results from Qiao et al., who reported weakened Raman signals in MoS_2_ QDs produced via a multi-step lithium intercalation process [14]. The reduced Raman signals in the F-MoS_2_ QDs can be attributed to multiple factors, including structural defects and charge transfer effects. The reduction in the Raman signal intensity may be due to the signals of the Raman-active bands falling below the resolution of the spectrometer used in this study, which had lower-magnification objective lenses compared to those in other studies [14].

Alternatively, the diminished Raman signals could result from structural defects in the MoS_2_ QDs. Defects such as in-layer disorder or the separation of the Mo and S monolayers caused by exfoliation can significantly reduce the intensity of Raman-active peaks [15]. Another possibility is that the absence of Raman-active peaks in the F-MoS_2_ QDs is due to quenching effects caused by 11-MUA functionalization, similar to the quenching observed in their fluorescence spectra [12].

Another potential reason is the charge transfer effects between the MoS_2_ QDs and the functionalizing molecules (11-MUA). Functionalization introduces electron-withdrawing or electron-donating groups, which can alter the phonon vibrational states of MoS_2_ QDs. Such charge transfer interactions can lead to Raman mode quenching by suppressing phonon lifetimes or modifying electron–phonon coupling, resulting in weaker Raman signals.

The Tauc method was used to extrapolate the band gap of the bare MoS_2_ QDs from the UV–Vis absorbance data, yielding a band gap energy of 3.70 eV. The band gap of the MoS_2_ quantum dots (QDs) is tunable due to quantum confinement effects. While bulk MoS_2_ has an indirect band gap of approximately 1.2–1.3 eV, MoS_2_ QDs exhibit a larger, direct band gap. The estimated band gap of QDs can vary significantly depending on the particle size and the solvent, with reported values reaching as high as 5 eV [16]. This variation is attributed to differences in the quantum dot size, as smaller diameters result in larger band gaps. The Tauc plot also shows a less steep curve than expected for a typical semiconductor crystal, suggesting structural or compositional deviations.

Dynamic light scattering (DLS) measurements indicated that both the bare and functionalized MoS_2_ QDs are exceedingly small, with estimated sizes below 1 nm in diameter. Considering the instrument’s margin error and comparisons with similar quantum dots, this suggests that their actual size could be 5–10 nm. The size of the QDs is consistent with other studies on hydrothermally synthesized defect-engineered QDs [17]. This size estimation aligns with the band gap determined via the Tauc method, as smaller QDs generally exhibit wider band gaps due to quantum confinement effects. For instance, similarly synthesized MoS_2_ QDs with slightly smaller diameters have been reported to exhibit band gaps of 4.30 eV, further supporting the observed size–band gap correlation [16]. The size distribution of QDs plays a critical role in their antibacterial activity. Smaller QDs exhibit enhanced passive diffusion into bacterial cells, improving cellular uptake and intracellular interactions. Additionally, size-dependent quantum confinement effects influence ROS generation, which is a key antibacterial mechanism. Larger nanomaterials, such as nanoflowers and nanosheets, tend to have significantly lower antibacterial activity compared to smaller QDs [18]. This is likely due to the reduced surface area-to-volume ratio, which limits the interaction with bacterial membranes and decreases the ROS production efficiency. Studies on carbon QDs suggest that an optimal size of ~3 nm is particularly effective against Gram-negative bacteria such as *E. coli*, as this size provides a balance between membrane penetration and ROS-mediated oxidative stress [19]. The hydrothermal method used to exfoliate the QDs from bulk material, while green and environmentally friendly, has poor size control, as indicated by the size distribution data from DLS. Nevertheless, the smaller F-MoS_2_ QDs synthesized in this study remain advantageous, as they maximize the bacterial interaction, penetration, and oxidative damage, contributing to their observed antibacterial effects [20].

The zeta potential data show that all of the quantum dots exhibit a neutral membrane potential (Table 1), which is expected for quantum dots with antibacterial activity. In the literature, a positive membrane potential is often associated with enhanced antibacterial effects, as it facilitates better adhesion to bacterial cell membranes and improves permeation [21].

The neutral zeta potential of the F-MoS_2_ QDs is also consistent with observations made during TEM imaging, which showed the aggregation of QDs. This is expected, as a neutral zeta potential correlates with decreased colloidal stability. The neutral membrane potential of the QDs is primarily attributed to the presence of Li^+^ ions from LiCl used during the hydrothermal exfoliation treatment. Typically, MoS_2_ QDs exhibit a negative membrane potential due to surface charge effects, which enhances their colloidal stability through electrostatic repulsion [22]. However, in this case, Li⁺ ions neutralized the surface charge, resulting in a neutral zeta potential. Despite the neutral zeta potential, the F-MoS_2_ QDs still exhibit antibacterial activity, suggesting that their mechanism does not solely rely on electrostatic interactions with bacterial membranes. While positive zeta potential can enhance bacterial adhesion and membrane permeation, alternative antibacterial pathways remain effective. The primary mode of action for F-MoS_2_ QDs appears to be reactive oxygen species (ROS) generation, which induces oxidative stress and cellular damage. This process does not necessarily require direct electrostatic interactions with bacterial membranes, as ROS can diffuse and exert antibacterial effects within cells, leading to organelle damage and DNA disruption [23]. Additionally, the functionalization of MoS_2_ QDs with 11-mercaptoundecanoic acid (11-MUA) may facilitate bacterial interactions through hydrophobic and Van der Waals forces, compensating for the absence of positively charged functional groups.

The antibacterial activity of F-MoS_2_ QDs is attributed to their surface functionalization rather than aggregation or physical changes in solution. While the neutral zeta potential of F-MoS_2_ QDs suggests reduced colloidal stability, the antibacterial activity observed in well-dispersed solutions at lower concentrations indicates that ROS generation and functional group interactions with bacterial membranes play a more significant role than aggregation-driven effects. The lack of antibacterial activity in bare MoS_2_ QDs, despite similar size and colloidal properties, further supports the functionalization-dependent nature of bacterial inhibition.

To evaluate the antibacterial activity, both optical density (OD600) measurements and spot counting on agar plates were employed. OD600 provides real-time monitoring of bacterial growth in liquid culture, allowing for the quantitative assessment of bacterial viability over time, while spot counting enables the direct visualization of bacterial colonies.

The growth inhibition testing was adapted from the method described by Spagnolo et al. using UV–Vis spectroscopy to monitor the optical density (OD) at 600 nm of the QD solutions at various concentrations after incubation with *Staphylococcus aureus* [24]. The results indicated that the bare MoS_2_ QDs did not exhibit any antibacterial activity (Table 2). In contrast, the F-MoS_2_ QD solution significantly reduced the OD at concentrations as low as 5.0 mg/mL, demonstrating substantial antibacterial activity at that concentration. Testing using spot counting on agar plates revealed bacterial colonies in solutions with concentrations below 10.0 mg/mL. Therefore, the minimum inhibitory concentration (MIC) of the F-MoS_2_ QDs was determined to be 10.0 mg/mL, which would not be sufficiently low for industrial antibacterial applications. While factors such as bacterial strain variability, QD aggregation, and culture medium composition could influence the spot counting results, the consistency of trends between OD600 and plate counting supports the reliability of our findings.

The zeta potential measurements confirm that phenylalanine was not present on the surface of the quantum dot, as it would provide a positive membrane potential. However, antibacterial activity was still observed, suggesting alternative functionalization strategies could enhance efficacy. Incorporating positively charged groups or using thiol-based ligands with enhanced stability can improve bacterial adhesion. Enhanced antibacterial effects are expected if positively charged groups (e.g., quaternary ammonium cations) are incorporated, as electrostatic interactions with bacterial membranes play a crucial role in antimicrobial activity. This highlights the need to optimize the synthesis and surface functionalization of MoS_2_ QDs to achieve more potent antibacterial activity at lower concentrations.

A recent study using more complex thiol ligand chemistry and positively charged surface functional groups has reported MIC values as low as 7 ppm for MRSA bacteria. They found that surface functionalization significantly influences bacterial selectivity, as leucine-functionalized MoS_2_ QDs were selective for Gram-positive bacteria, whereas phenylalanine-functionalized MoS_2_ QDs exhibited broad-spectrum antibacterial activity against both Gram-positive and Gram-negative bacteria.

Moreover, increasing colloidal stability could further enhance the antibacterial effectiveness. For instance, using lipoic acid instead of 11-MUA could provide stronger anchoring to the MoS_2_ QD surface due to its two thiol groups, potentially improving long-term dispersion and reactivity [25]. When compared to other nanomaterial-based antibacterial agents, optimized F-MoS_2_ QDs exhibit promising performance. For example, silver nanoparticles (5 nm in diameter), another widely studied antibacterial nanomaterial, have an MIC of 70 ppm against *Staphylococcus aureus* [26]. Vancomycin has an MIC of 0.5–2 ppm for MRSA, but, like other antibiotics, the antibiotic resistance remains a major concern [27]. While MIC values can vary depending on bacterial strain differences, these results suggest that MoS_2_ QDs hold significant potential as an alternative antibacterial agent to traditional antibiotics.

## 4. Conclusions

A one-step hydrothermal synthesis method involving lithium intercalation offers a simple and green approach for producing MoS_2_ QDs from bulk MoS_2_ powder. Antibacterial testing demonstrated that functionalizing MoS_2_ QDs with 11-MUA provided effective antibacterial activity at concentrations as low as 10 mg/mL. Future research should focus on determining the antibacterial mechanism of the functionalized MoS_2_ QDs using ROS assay kits and bacterial staining tests, as well as conducting long-term toxicity and stability testing to assess their behavior under real-life conditions. While MoS_2_ QDs are reported to be non-cytotoxic compared to bulk MoS_2_, further in vivo studies will be necessary to confirm their safety in biomedical applications, particularly for coatings on medical implants, catheters, and wound dressings [28]. Compared to silver nanoparticles, which have documented neurotoxicity, MoS_2_ QDs offer a promising alternative for antibacterial coatings in healthcare settings.

However, scalability remains a challenge. The hydrothermal exfoliation method used in this study, while green and cost-effective, has limitations, such as a low yield, high water consumption, and aggregation issues due to LiCl intercalation, which affects the colloidal stability. Additionally, while many studies use Na_2_MoO₄ as a precursor, it is significantly more expensive than bulk MoS_2_ and requires acidic conditions, making large-scale production more costly.

To improve the industrial feasibility, further optimization of functionalization strategies is required. Functionalizing MoS_2_ QDs with positively charged groups (e.g., quaternary ammonium cations and cationic peptides) or thiol-based ligands (e.g., lipoic acid) could enhance the antibacterial efficacy while maintaining biocompatibility. Additionally, long-term stability studies should be conducted to evaluate the performance of MoS_2_ QDs in physiological environments (e.g., exposure to bodily fluids, sterilization procedures, and mechanical wear). These studies will be crucial in determining the viability of F-MoS_2_ QDs as durable antimicrobial coatings for medical devices.

## Figures and Tables

**Table 1:** The membrane potential of bare MoS_2_ and F-MoS_2_ QDs based on zeta potential measurements.

pH	Bare MoS_2_ QDs (mV)	F-MoS_2_ QDs (mV)
3	4.36	1.85
7	1.47	8.35
11	11.38	12.22

**Table 2:** Optical density values at 600 nm for growth inhibition testing against the *Staphylococcus aureus* with bare MoS_2_ QDs and F-MoS_2_ QDs.

Concentration (mg/mL)	Bare MoS_2_ QDs	F-MoS_2_ QDs
Blank	0.155	0.155
Control	0.678	0.844
0.5	0.789	1.024
1.0	0.736	0.814
2.5	0.663	0.629
5.0	0.710	0.319
10.0	0.785	0.277
25.0	0.918	0.261
50.0	0.743	0.263

## Data Availability

The data supporting this study are included in the Supplementary Materials.

## Supplementary Materials

The following supporting information can be downloaded at: https://www.mdpi.com/article/10.3390/ma18061352/s1, 1. Synthesis of MoS_2_ QDs; Scheme S1. Flow chart of the synthesis of MoS_2_ quantum dots by hydrothermal treatment; 2. Functionalization of MoS_2_ QDs; Scheme S2. Illustration of the reaction scheme for the functionalization of the quantum dots by phenylalanine; 3. Characterizations; 4. Antibacterial testing; 5. Alternative synthesis; Figure S1. (a) UV–Vis spectra, (b) Fluorescence spectra of alternate synthesis methods; Figure S2. Full Raman spectra of the bare MoS_2_ quantum dots; Figure S3. Raman spectra of the functionalized quantum dots from 350 to 430 cm^−1^.

## Abbreviations

The following abbreviations are used in this manuscript:

MDPI	Multidisciplinary Digital Publishing Institute
DOAJ	Directory of Open Access Journals
QD	Quantum dots
ROS	Reactive oxygen species
F-MoS2	Functionalized MoS2
11-MUA	11-mercaptoundecanoic acid
MIC	Minimum inhibitory concentration
