# Supplementary material for: Antibacterial Coating Based on Functionalized MoS2 Quantum Dots
Source: Materials (Basel). 2025 Mar 19;18(6):1352. doi: 10.3390/ma18061352 (PMC11943794; doi:10.3390/ma18061352)
Supplement: Supplementary file 1 [file materials-18-01352-s001.zip › materials-3503083-supplementary.pdf]

Supplementary Information

Antibacterial Coating Based on Functionalized MoS_2 Quantum Dots

Toby Chan, [a] Soha Ahmadi, [a] Zahra Ramezani,[*a,b] and Michael Thompson [*a]

[a] Department of Chemistry, University of Toronto, 80 St George St, Toronto, ON M5S 3H6, Canada;

[b] Toxicology Research Center, Medical Basic Sciences Research Institute, Ahvaz Jundishapur University of Medical Sciences, Ahvaz, Iran

Experimental

1. Synthesis of MoS_2 QDs.

A hydrothermal method involving lithium intercalation was utilized for the exfoliation of the quantum dots from bulk material. In a typical preparation process, 1.69 mmol MoS_2 bulk powder (0.27 g) and 0.71 mmol LiCl (30 mg) were dissolved in 60 mL of a 10% ethanol solution in an 80 mL Teflon-lined stainless-steel autoclave. The mixture underwent hydrothermal treatment at 200 C for 22 h and was allowed to cool to room temperature naturally prior to separation. The supernatant containing the MoS_2 quantum dots was collected using gravity filtration and then centrifuged at 2000 rpm for 30 minutes to separate the product from the unreacted bulk material. The supernatant collected was a transparent, dark grey solution. A UV lamp was used to partially confirm the synthesis was successful, as the bulk material does not have any visible fluorescence, whilst the quantum dots have a faint blue glow when irradiated by a UV lamp. The supernatant was dried in the oven to obtain dried quantum dots. The dried quantum dots were a light brown-grey film around the scintillation vial and formed a dark grey hygroscopic solid when scraped and collected. Scheme S1 shows the flow chart for the synthesis of the MoS_2 quantum dots by hydrothermal synthesis.

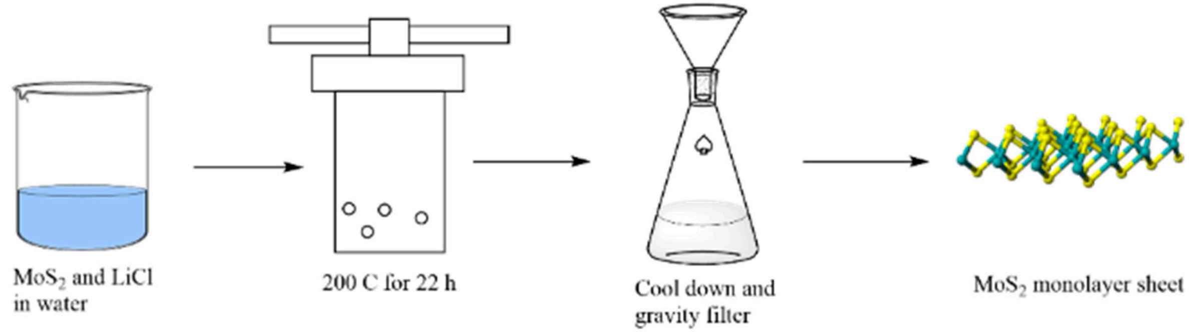

Scheme S1. Flow chart of the synthesis of MoS_2 quantum dots by hydrothermal treatment.

2. Functionalization of MoS_2 QDs

For surface functionalization, the 0.19 mmol of the dried MoS_2 quantum dots (0.03 g) were dissolved in a scintillation vial with 5 mL of deionized water to yield a 15 mg/mL solution. The solution was then combined with 0.046 mmol 11-mercaptoundecanoic acid (11-MUA, 10 mg) and reacted for 20 minutes with stirring. The pH of the solution was checked to ensure a pH between 5 and 6. 0.01 mmol EDC (1.6 mg) and 0.021 mmol NHS (2.4 mg) were added to the solution and allowed to react for 15 minutes with stirring. After reacting, of 0.11 mmol 2-mercaptoethanol (8 μL) was added to the reaction vessel to quench the remaining, unreacted EDC. 0.041 mmol phenylalanine (6.8 mg) was dissolved in 2 mL of water and added in half portions to the solution mixture, before being allowed to react for 3 hours with stirring. The functionalized quantum dot (Scheme S2) precipitate was separated from the supernatant using a 10 kDa centrifuge filter at 12 000 rpm for 1 h. Once the precipitate was separated, it was redispersed in water to allow for recovery. The solution mixture was lyophilized to give a stable grey-blue powder prior to antibacterial testing.

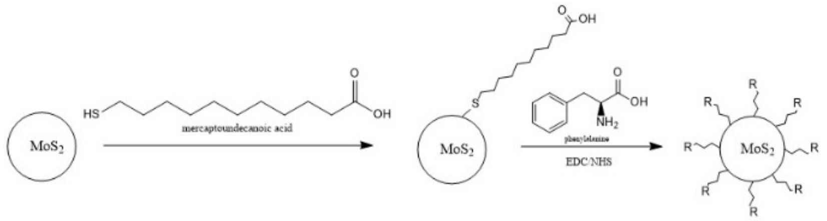

Scheme S2. Illustration of the reaction scheme for the functionalization of the quantum dots by phenylalanine.

3. Characterizations

The quantum dot samples were characterized using UV-Vis, fluorescence, IR, Raman, and fluorescence microscopy. The UV-Vis absorbance experiments were conducted on a UV-1600PC spectrometer (VWR International, Mississauga, Ontario) scanning from 200 to 750 nm using a standard 10 mm path length quartz cuvette. The fluorescence spectroscopy experiments were conducted using a PerkinElmer FL6500 fluorescence spectrometer operating with a slit width of 5 mm using a standard 10 mm path length quartz micro-cuvette. The IR spectroscopy experiments were conducted using a PerkinElmer Spectrum Two FTIR equipped with a $LiTaO_3$ detector scanning from 4000 to 500 cm^{-1}. The Raman spectroscopy experiments were conducted using a Bruker SENTERRA Dispersive Raman Microscope with a 20 mW, 532 nm laser for excitation, 0.1 mm aperture, and a 20x objective lens. The fluorescence microscopy images were taken using the 40x objective lens of an OMAX 40X 2500X epifluorescence microscope using a blue excitation light.

4. Antibacterial Testing.

The growth inhibition testing was adapted from a method by *Spagnolo et al.* For the preparation of the samples being analyzed, a blank, control, and solutions containing a range of different quantum dot concentrations, from 50 mg/mL to 0.5 mg/mL, were prepared prior to the deposition of bacteria. Each sample container was sterilized in the microwave prior to sample preparation. The blank was prepared using lysogeny broth (LB) and water. The samples, excluding the blank, were spiked with a pipette tip of *Staphylococcus aureus* and then incubated overnight on a tilt tray at 37 C. The samples were measured using a UV-1600PC spectrometer (VWR International, Mississauga, Ontario) to measure the optical density of the solutions at 600 nm (OD600).[14]

For spot counting testing, the Petri dishes were prepared from melted agar, set in the Petri dishes, and allowed to cool. Each solution from the growth inhibition testing was spotted onto the agar plates (10 µL x 3) and allowed to incubate at 37 C overnight.

5. Alternative synthesis

The alternative synthesis involving sodium molybdate as the source of molybdenum was adapted from a method by *Wang and Ni*. The quantum dots were prepared using hydrothermal treatment by using sodium molybdate and L-cysteine as precursors. 0.1 g of Na_2MoO4 was dissolved in 10 mL of water. The sodium molybdate solution was then sonicated for 5 minutes and adjusted to 6.5 pH using 1M HCl. After pH adjustment, 0.2 g of L-cysteine was added along with 20 mL of water, before sonicating for 10 minutes. The solution was transferred to a Teflon-lined stainless-steel autoclave and reacted at 200 C for 36 h. The resulting solution after hydrothermal treatment was an opaque, dark black solution. After reacting, the solution was allowed to cool naturally and the

supernatant containing the MoS_2 quantum dots was separated from the particulate via centrifugation at 12 000 rpm for 1 h. The resulting supernatant solution was a transparent bright orange solution.[6]

For the alternative synthesis involving the residential microwave, bulk MoS_2 powder was used as the precursor source for MoS_2. The quantum dots were prepared using hydrothermal treatment involving lithium intercalation. 0.3 g of MoS_2 powder was combined with 0.1 g of LiCl and dissolved in 20 mL of a 10% ethanol aqueous solution in an Erlenmeyer flask. The mixture underwent hydrothermal treatment in the microwave at full power in 30-second increments until the target reaction time was reached, with an additional 10 mL of ethanol added every 10 minutes to prevent the reaction vessel from drying out. Reaction times from 1 minute to 30 minutes were tested using this method. The supernatant containing the MoS_2 quantum dots was gravity-filtered and centrifuged at 2000 rpm for 30 minutes to separate it from the unreacted bulk material. The resulting supernatant collected was a transparent, dark grey solution.

UV-Vis and fluorescence spectroscopy were used to confirm the synthesis of quantum dots using the two alternative methods. Before any characterization work using instruments, the solutions were inspected using a UV lamp for fluorescence, which both exhibited a faint blue glow as emission upon irradiation. Two attempts were made to dry the sodium molybdate synthesized samples, one using the oven and one using a lyophilizer. The attempt to dry the sodium molybdate synthesized sample resulted in the production of an opaque, black solution that could not be separated using centrifugation. The attempt to dry the sodium molybdate synthesized sample using lyophilization yielded a waxy orange film around the scintillation vial that could not be reconstituted using water.

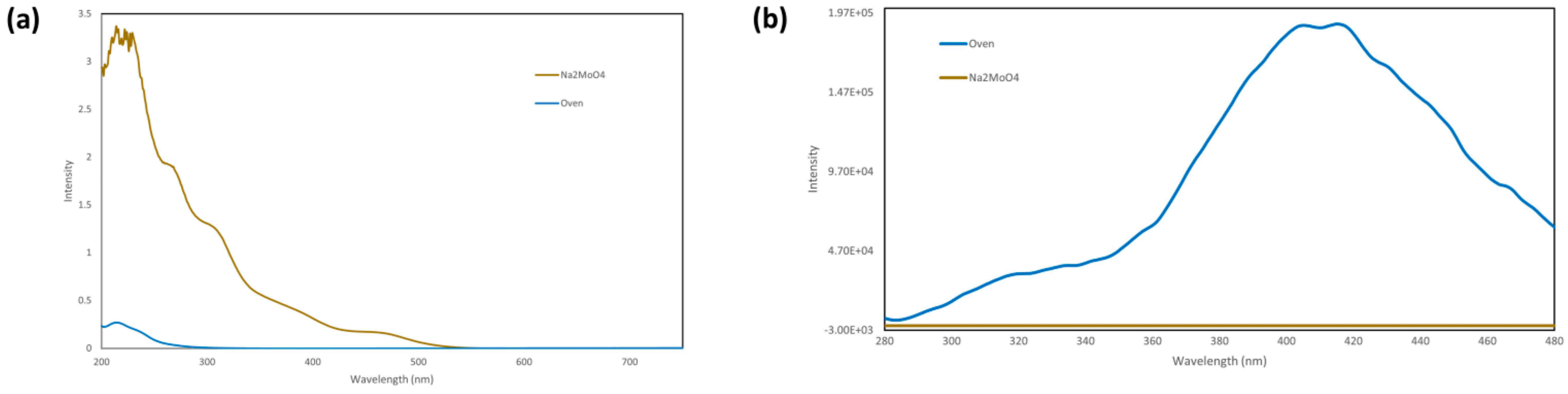

Figure S1. a) UV-vis spectra, b) Fluorescence spectra of alternate synthesis methods.

The sodium molybdate synthesized quantum dots have a significantly higher absorbance than the microwave or oven-synthesized quantum dots, even when dilution was considered for the solutions. More dilute solutions of the sodium molybdate sample also yielded much higher absorbances than the other methods using MoS2 powder. However, the UV-Vis data does not conclusively determine whether or not the quantum dots were successfully synthesized. Initially, from the UV-Vis spectra, the microwave synthesis appeared to be successful due to the close similarities exhibited between the UV-Vis absorbance spectra of the microwave-synthesized samples and the oven-synthesized samples. Both the microwave and oven-synthesized samples exhibited absorbance maximum peaks at 205 nm. The fluorescence spectrum is much more conclusive in determining the success of the synthesis method, as the quantum dots are established as exhibiting a broad fluorescent emission peak from prior characterization. Comparing the fluorescence spectra of the different synthesis methods, the oven synthesis sample shows a visible, broad emission peak with a very high fluorescence intensity at around 415 nm, whilst the microwave and sodium molybdate samples show no visible emission peaks. Based on the absence of emission peaks, both the microwave and sodium molybdate synthesis methods were both unsuccessful in synthesizing MoS_2 quantum dots.

In conclusion, the data shows that a one step synthesis involving lithium intercalation provides a simple, green method for the preparation of MoS_2 quantum dots from bulk MoS_2 powder. The results from the antibacterial testing confirmed that when functionalized, even with a less potent antibacterial functional group like 11-MUA, the functionalized MoS_2 quantum dots still exhibited effective antibacterial activity at concentrations as low as 10 mg/mL.

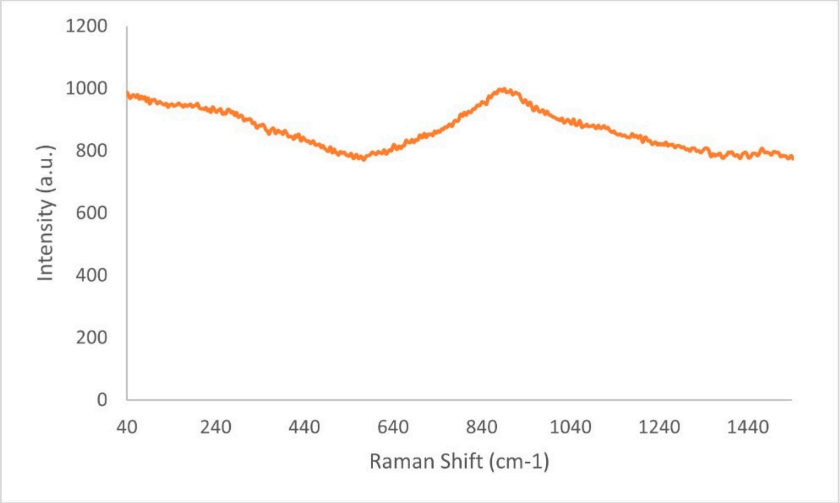

Figure S2. Full Raman spectra of the bare MoS_2 quantum dots.

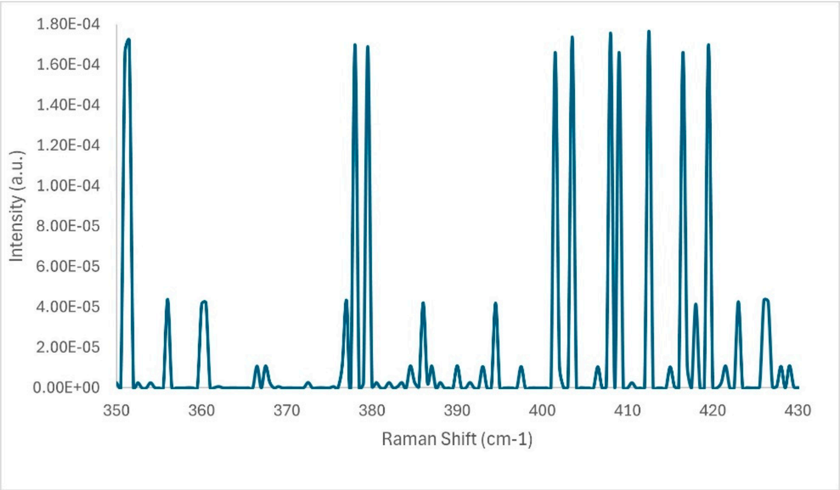

Figure S3. Raman spectra of the functionalized quantum dots from 350 to 430 cm^{-1}.
